# Cellular Senescence in Renal and Urinary Tract Disorders

**DOI:** 10.3390/cells9112420

**Published:** 2020-11-05

**Authors:** Yohan Santin, Philippe Lluel, Pascal Rischmann, Xavier Gamé, Jeanne Mialet-Perez, Angelo Parini

**Affiliations:** 1 Institut des Maladies Métaboliques et Cardiovasculaires, Inserm, Université Paul Sabatier, UMR 1048—I2MC, 31432 Toulouse, France; yohan.santin@inserm.fr (Y.S.); jeanne.perez@inserm.fr (J.M.-P.); 2 Urosphere SAS, Rue des Satellites, 31400 Toulouse, France; philippe.lluel@urosphere.com; 3 Department of Urology, Kidney Transplantation and Andrology, Toulouse Rangueil University Hospital, 31432 Toulouse, France; rischmann.p@chu-toulouse.fr (P.R.); game.x@chu-toulouse.fr (X.G.)

**Keywords:** senescence, aging, SASP, chronic kidney disease, urogenital disorders

## Abstract

Cellular senescence is a state of cell cycle arrest induced by repetitive cell mitoses or different stresses, which is implicated in various physiological or pathological processes. The beneficial or adverse effects of senescent cells depend on their transitory or persistent state. Transient senescence has major beneficial roles promoting successful post-injury repair and inhibiting malignant transformation. On the other hand, persistent accumulation of senescent cells has been associated with chronic diseases and age-related illnesses like renal/urinary tract disorders. The deleterious effects of persistent senescent cells have been related, in part, to their senescence-associated secretory phenotype (SASP) characterized by the release of a variety of factors responsible for chronic inflammation, extracellular matrix adverse remodeling, and fibrosis. Recently, an increase in senescent cell burden has been reported in renal, prostate, and bladder disorders. In this review, we will summarize the molecular mechanisms of senescence and their implication in renal and urinary tract diseases. We will also discuss the differential impacts of transient versus persistent status of cellular senescence, as well as the therapeutic potential of senescent cell targeting in these diseases.

## 1. Overview of Cellular Senescence

Cellular senescence refers to a state of stable cell cycle arrest that can be initiated by various stresses despite the presence of growth-promoting stimuli. Senescence can occur in multiple contexts across tissue and organ lifespans. Within this line, acute senescence generated early in life provides physiologically-appropriate responses in the developing embryo during organogenesis. This transient phenotype also plays key roles in tissue homeostasis, wound healing, and regeneration. In addition, senescence-related growth arrest prevents tumorigenesis and neoplastic transformation [1,2]. Conversely, chronic accumulation of senescent cells is increasingly recognized as a driver of various features of aging, including age-related diseases and tissue deterioration [3].

**Figure 1:**
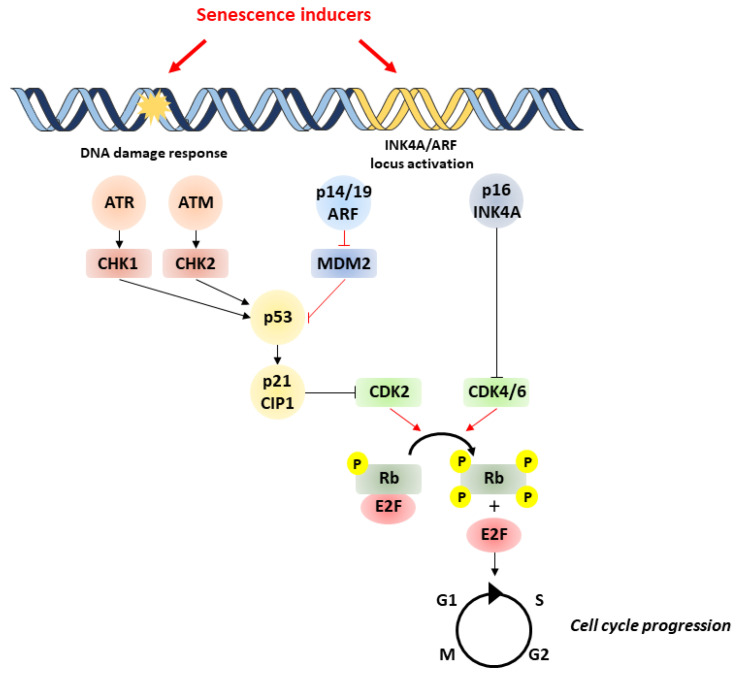
Pathways involved in cell senescence. Senescence is induced by various stressors, which trigger DNA damage and subsequent activation of p53/p21 and p16/pRb pathways. p53 activation is achieved by phosphorylation by ATM/ATR and checkpoint kinases Chk1/Chk2. Likewise, p53 activity can be increased by binding of p14/P19^ARF^ product of the INK4a locus to MDM2 preventing degradation of p53. p53 can induce senescence by activating p21, which inhibits CDK2, leading to the hypophosphorylation of Rb. In addition to p53, the accumulation of the tumor-suppressor p16^INK4A^ also leads to cell cycle arrest through the inhibition of CDK4/CDK6 and subsequent hypophosphorylation of Rb. This enables Rb to bind to E2F, inhibiting cell cycle progression. ATM, ataxia-telangiectasia-mutated kinase; ATR, ataxia-telangiectasia- and ATM-Rad3-related kinase; MDM2, murine double minute 2; Rb, retinoblastoma.

Major triggers of senescence include repeated cell division and telomere shortening, first described by Leonard Hayflick in 1961 (now referred to as replicative senescence), but also stressors such as oncogenic mutations, metabolic and oxidative stresses, mitochondrial dysfunction, and inflammation (referred to as stress-induced premature senescence or SIPS) [4]. Senescent cells are often characterized by a persistent DNA damage response (DDR) and the triggering of cell signaling cascades involved in DNA repair and cell cycle arrest, including chronic ATM (ataxia-telangiectasia-mutated) or ATR (ataxia-telangiectasia- and Rad3-related) kinase activation. A typical example of the consequences of DNA damage is the generation of the phosphorylated form of the H2A histone family member X (γH2AX) by ATM. These pathways converge to cell cycle arrest and senescence, through activation of p53/p21^CIP1^ and p16^INK4A^ that inhibit cyclin-dependent kinases (CDKs) and retinoblastoma protein (RB), enhancing checkpoint activity and inducing G1/S (and occasionally G2/M) cell cycle arrest [5] (Figure 1). 

Despite the cell cycle arrest, senescent cells remain metabolically active, releasing a particular secretome that can affect neighboring cells and ultimately tissue function. This phenotype is defined as “senescence-associated secretory phenotype” (SASP) and relies on the production of a specific “senescence-messaging secretome” (SMS) including pro-fibrotic and pro-inflammatory factors like IL-1β, IL-6, TGFβ, PAI-1, or CCN2, which can act in a paracrine and autocrine fashion [6,7]. Although pro-inflammatory cytokines and chemokines are relatively conserved SMS components, SMS composition can vary depending on the biological context, often reflecting both the origin of the senescent cells and the initiating stimuli. Therefore, the functions attributed to the SMS are very diverse and depend on the transient versus persistent status of senescent cells, the nature of the SMS, and the surrounding environment of the cells subjected to the senescent secretome. For example, by an autocrine mechanism, the SMS reinforces the senescence-associated growth arrest by implementing a positive-feedback loop, leading to persistence and propagation of senescence within tissues [8]. On the other hand, the pro-inflammatory nature and inflammatory mediators of the SMS are powerful drivers of tumor progression through a paracrine mechanism. 

Other key features of senescent cells, including morphological and metabolic changes, have been described. Indeed, in addition to enlarged size, flattened shape, and sometimes multiple or enlarged nuclei, senescent cells exhibit striking chromatin changes, through the formation of senescence-associated heterochromatic foci (SAHFs). These senescence-specific heterochromatic compartments are enriched in chromatin modifications and sequester genes implicated in cell-cycle control, reinforcing the senescence-associated growth arrest. Of note, genomic regions contained in the SAHFs are found in lamina-associated domains (LADs) [9]. Upon induction of senescence, lamin B1 is downregulated, making these LADs detach from the nuclear periphery and cluster within the nuclei, participating in the disruption of nuclear envelope integrity. In addition to nuclear modifications, senescent cells usually display extensive vacuolization due to an enhanced lysosomal content, reflected by increased levels of β-galactosidase activity. This enzymatic activity is found in non-senescent cells under physiological conditions (pH 4.0–4.5), but it is significantly increased in senescent cells and detectable at pH 6.0 (suboptimal conditions). Importantly, senescence-associated β-galactosidase (SA-β-gal) activity can be measured both in vitro and in vivo, and is considered a key hallmark of senescence [10]. Of note, lipofuscin, an aggregate of oxidized proteins, lipids, and metals that are known to accumulate in aged tissues, was recently reported to co-localize with SA-β-gal activity in senescent cells, and could represent another easily measurable marker of senescent cells [11].

Metabolic changes displayed by senescent cells include increases in glycolysis, mitochondrial metabolism, and autophagy dysfunction, while their roles remain controversial. For instance, the production of SASP components has been shown to rely on enhanced ATP production mediated by increased mitochondrial metabolism and glycolysis, but finally leading to a massive proteotoxic stress [12]. Interestingly, the latter can be attenuated by activating autophagy. On the other hand, some studies have shown that inhibition of autophagy facilitates senescence [13]. Within this line, mitochondrial autophagy (mitophagy) is usually decreased in senescent cells, resulting in an altered mitochondrial network, accumulation of dysfunctional mitochondria, and ROS-induced senescence, contributing to aging-related metabolic dysfunction [14,15]. 

An increase in senescent cell burden has been associated with age-related disorders, chronic diseases, and accelerated mortality [16]. Within this line, cellular senescence has been linked to obesity-related metabolic derangements [17], chronic lung diseases [18], cardiovascular aging [19], as well as neurodegenerative disorders like Alzheimer’s [20] and Parkinson’s [21]. Recently, senescence has drawn a particular attention in the field of urogenital disorders, which affect the urinary tract or reproductive organs, and have a higher incidence in the elderly population. Indeed, studies reported an increase in senescent cell burden in renal diseases and in aging-associated lower urinary tract symptoms [22,23,24,25,26]. However, the role of cellular senescence in such disorders is still unclear. In this review, we will discuss the molecular mechanisms underlying cellular senescence and their implication in renal and urogenital diseases.

## 2. Senescence in the Kidneys

Renal aging is characterized by a loss in renal mass (cortical glomeruli and tubules) and impaired renal function, as well as various histological changes including glomerulosclerosis, tubulointerstitial fibrosis, and nephrosclerosis [27]. These phenotypic changes are associated with a decline of kidney function and diminished proliferative response of tubular cells (and to a lesser extent of glomerular and interstitial cells), which correlate with markers of cellular senescence such as p16^INK4A^, SA-β-gal, and telomere shortening [22,23]. Age-associated changes of the kidney are important not only because normal aging alters renal function, but also because of the high frequency of renal diseases in the elderly population. In particular, chronic kidney disease (CKD), defined by the persistent loss of kidney function, currently affects about 13.4% of the global population [28]. Importantly, CKD is regarded as an accelerated aging of the kidney and an independent risk factor for cardiovascular events in the elderly, often leading to end-stage renal disease (ESRD) [29]. Of note, similar pathogenic mechanisms are involved in both aging and CKD onset, such as an increase in senescent cell burden as well as the secretion of pro-fibrotic and pro-inflammatory factors. This latter feature, known in chronic renal failure as CKD-associated secretory phenotype (CASP), shares many similarities with SASP regarding the secreted factors, which may be some mediators of the crosstalk between cellular senescence and CKD [30,31]. 

### 2.1. Acute Renal Injuries

Acute damage of tubular epithelial cells like ischemia-reperfusion injury (IRI) or acute kidney injury (AKI) are primary drivers of renal injury and CKD, especially in elderly patients who have an increased propensity to develop progressive CKD [32]. Notch-induced senescent state in proximal tubules triggers maladaptive repair and leads to lower outcome of old kidneys after IRI [33]. Within this line, mouse models of telomerase deficiency, with higher telomere shortening and accelerated senescence, exhibited delayed renal tubular regeneration and increased apoptosis after IRI [34]. Furthermore, p16^INK4A^ knockout mice show increased epithelial proliferation and functional recovery after IRI, meaning that post-injury renal recovery is improved by lowering the number of senescent cells and improved regenerative capacity of aged kidneys [35]. This idea is further supported in AKI, where kidneys with a high content of senescent cells are more prone to develop CKD due to incomplete recovery and development of tubulointerstitial fibrosis [36]. Nevertheless, some conflicting results highlighted the more intricate role of cell senescence in kidney injury. This is well illustrated by the higher susceptibility of p21 knockout mice to ischemia-induced acute renal failure [37]. Similar results were observed in IRI, where senescence induction by CDK4/CDK6 inhibition ameliorated kidney function following injury [38]. 

Overall, these observations support a dual role of senescent cells in response to acute renal injuries. One could hypothesize that activation of senescence during an acute stress would limit damage propagation by transiently decreasing cell cycle activity, thereby lessening kidney impairment and mortality. However, if senescent cells accumulate after the stress, it is likely that they negatively influence local milieu and further deteriorate organ function. In any case, further studies are needed to better characterize the roles of senescent cells at various time points and in different models of acute renal injuries.

### 2.2. Chronic Renal Diseases

ESRD is the most common and serious consequence of diabetes, a pathological condition where damage to the kidneys usually occurs over many years. Importantly, diabetic nephropathy and chronic renal disease are characterized by a progressive onset of fibrosis together with a persistent and low-grade inflammation, both of them being hallmark features of senescent cells and related SASP. Of note, kidneys with type 2 diabetic nephropathy displayed an accelerated senescent phenotype in tubule cells and, to a lesser extent, podocytes, as indicated by increased p16 expression and SA-β-gal activity [39]. A similar senescent phenotype was observed when proximal tubule cell cultures were incubated under high-glucose media [39]. Accordingly, streptozotocin-induced hyperglycemia increased senescent cell burden in mouse kidneys in the early stage of type 1 diabetes, characterized by an increase in the renal expression of p21 and SA-β-gal staining in tubular epithelial cells, as well as the acquisition of SASP in endothelial cells and macrophages [40,41]. Interestingly, SASP mediators were also elevated in serum of patients in early stage diabetic nephropathy [42]. An impaired immune system (e.g., due to aging) may cause senescent cells to escape immune clearance and further maintain a senescent secretome [43]. SMS components could therefore constitute a major source of inflammatory factors in diabetes, likely contributing to fueling the low-grade inflammation commonly observed in this pathology. Importantly, the clearance of senescent cells in a mouse model of obesity-induced metabolic dysfunction improved renal podocyte function, reduced microalbuminuria, and lowered circulating inflammatory mediators [17]. Overall, these results suggest that hyperglycemia is a key driver of senescence, the latter being a potential contributor to the development of diabetic nephropathy and consequent ESRD.

A major feature in ESRD is kidney fibrosis, which develops from a variety of diseases and leads to loss of renal function. Senescence is associated with renal disease progression, and accelerated tubular cell senescence leads to maladaptive repair and contributes to the pathogenesis of renal fibrosis [36]. Interesting data indicated that aged mice 6 weeks post-reperfusion had extensive tubulo-interstitial fibrosis and leukocyte infiltration, which correlated with increased p53 expression and SA-β-gal positive cells in kidney tubules [44]. In addition, AKI-induced senescence of proximal tubular cells produce pro-fibrotic growth factors that are capable of stimulating fibroblast proliferation and collagen production [45]. In human proximal tubular epithelial cell line and primary renal tubular cells, Wnt9a upregulated the levels of p16^INK4A^, p19^ARF^, p53, and p21, and induced TGF-β1 production by senescent tubular cells, finally promoting proliferation and activation of kidney fibroblasts. Notably, Wnt9a-induced renal fibrosis was inhibited by silencing of p16^INK4A^ in an IRI mouse model [46]. These results strengthen the general concept that senescent cells that appear after renal injury contribute to a pro-fibrotic and pro-inflammatory milieu through their SASP components, further promoting the gradual accumulation of renal fibrosis [47]. Nevertheless, contrary to the well-described detrimental effect of cellular senescence, there is evidence regarding a beneficial role of senescent cells in renal fibrosis. Within this line, Wolstein and coworkers have shown that p16^INK4A^ knockout mice spontaneously exhibited mesangial cell proliferation, myofibroblast differentiation, and increased matrix deposition in kidneys [48]. In addition, after unilateral ureteral obstruction, p16^INK4A^ knockout mice had enhanced tubular and interstitial cell proliferation, lower collecting duct apoptosis, greater collagen and fibronectin deposition, and no SA-β-gal staining compared with WT mice. These results suggest that p16^INK4A^ regulates cell proliferation and limits matrix production, thereby mitigating post-injury fibrosis [48]. Altogether, these discrepancies suggest a dual role of cellular senescence in the onset of renal fibrosis, meaning that further studies will be needed to elucidate the mechanisms by which senescent tubular cells promote or prevent matrix deposition.

As stated before, CKD can lead to ESRD if the renal function gradually deteriorates. In this case, alternative therapies such as kidney transplant are necessary for patients with ESRD. Studies on humans have correlated the levels of pre-transplant senescent cells in the kidneys with subsequent chronic allograft nephropathy [49,50]. Furthermore, in experimental rat models of transplant rejection, acute rejection was characterized by telomere shortening and consequent p21 and p16 overexpression, while SA-β-gal staining was found in tubular epithelial cells of allografts with chronic rejection [51]. Conversely, p16^INK4A^ deletion in mice led to improved renal function and resulted in superior recipient survival in a life-supporting transplant model [52]. Data in humans showed that telomere length in biopsies collected in the peri-transplant period predicted the long-term kidney allograft function, and telomere shortening was linked to complications of kidney transplantation including delayed graft function, acute rejection, and chronic allograft dysfunction [53]. Of note, senescence may also explain some of the impacts of age on graft outcome. Indeed, kidney transplants from aged donors exhibit a higher number of senescent cells and have a lower graft survival compared to “young kidneys” [54]. In addition, increased numbers of senescent cells in an older graft reduce the regenerative capacity of the transplanted kidney in response to injury [55]. Altogether, these results support a crucial role of senescence in kidney transplant, and the level of senescence before transplantation has been shown to be a good predictor of postoperative kidney function, suggesting that biological age is an important prognostic determinant for renal transplant outcome [56].

### 2.3. Cancers

Senescence induction by oncogene activation has long been recognized as a potent tumor-suppressive mechanism, thereby preventing the expansion of damaged and pre-neoplastic cells [57]. In line with this, deletion of the Apc tumor suppressor gene induces kidney senescence which inhibits the formation of renal carcinomas [58]. Interestingly, in addition to Apc deletion, loss of p21 or p16^INK4A^ was required to initiate renal carcinoma, supporting the anti-neoplastic role of senescence [58]. Besides, a recent study showed that calcitriol-induced histone demethylase JMJD3 increased p16^INK4A^ expression and cell senescence on human renal cancer cells in vitro, consequently promoting the anticancer activity of calcitriol [59]. Of note, renal cell carcinomas are associated with Von Hippel–Lindau (VHL) disease, an autosomal-dominant disorder caused by germline mutations of the VHL tumor-suppressor gene. Surprisingly, acute VHL inactivation has also been described as a trigger of HIF-independent senescence program mediated by Rb and p400 [60]. Interestingly, decreased p400 expression in renal cell carcinoma patients was associated with advanced tumor stage, higher grade of malignancy, and regional lymph node metastasis [61]. Together, these data suggest that the highly proliferative, decreased-p400 subgroup of renal cell carcinomas represents tumors that are characterized by a loss of senescence, making senescence an efficient tumor-suppressive mechanism that must be overcome to develop VHL-associated neoplasia. Importantly, senescence can also act as a driver of cancer development by altering the cellular microenvironment, mainly through the SMS components [62,63]. However, whether cellular senescence can favor tumor progression in renal cancers remains to be determined.

## 3. Senescence in the Prostate

The prostate is a small male gland with high incidence of disease concomitant with aging. Benign prostate hyperplasia (BPH), also known as prostate gland enlargement, is a very common disorder affecting about 50% men between 50 and 60 years old, and up to 90% men by age 85 [64]. In this case, the growth of the prostate gland to an unhealthy size may lead to uncomfortable urinary symptoms including dysuria, urgency, frequency, and nocturia, which significantly decrease patients’ quality of life [65]. Besides BPH, prostate cancer (PCa) is a common type of prostate disease among men and a leading cause of cancer-related deaths. While early prostate cancer does not usually cause symptoms, as cancer grows it may cause obstructive symptoms or pain related to a metastatic evolution. Interestingly, recent works highlighted the determining role of senescent cells and SASP in prostate disorders, opening up new avenues in the treatment of these common diseases.

### 3.1. Benign Prostate Hyperplasia (BPH)

Benign prostate hyperplasia epithelium is characterized by an enrichment of senescent cells [25]. Interestingly, epithelial and stromal human prostate cells exposed to ionizing radiation acquired a senescent phenotype, evidenced by increased SA-β-gal positive cells, enhanced p16^INK4A^ expression, and the acquisition of a pro-inflammatory and pro-fibrotic SASP [66]. Importantly, conditioned media from SASP-induced cells stimulated proliferation of non-irradiated prostatic epithelial cells by activating STAT5, ERK1/2, and AKT [66]. Among SASP components, IL-1α, and IL-8 have drawn a particular attention as key regulators of prostatic cell growth. Indeed, functional studies indicated that IL-1α can result in increased benign tissue growth in both xenograft and transgenic model systems in vivo [67]. This effect has been attributed, at least in part, to the IL-1α-mediated induction of stromal FGF-7 expression, that can act as a paracrine factor to stimulate the proliferation of non-senescent epithelial cells [68]. Alternatively, IL-1α can also induce IGF-1 in prostatic stromal fibroblasts, further enhancing the epithelial growth response [69]. Similarly, IL-8 has been reported to stimulate epithelial and stromal growth directly or through FGF-2 induction [70,71]. Overall, these results support the idea that cellular senescence and derived SASP of prostatic epithelial cells participate to the pathogenesis of BPH.

### 3.2. Prostate Cancer (PCa)

Prostate cancer (PCa) is a disease characterized by a markedly increased incidence with age. PCa progression relies on circulating androgens, and the initial step in managing advanced PCa is androgen-deprivation therapy (ADT). Although a subset of androgen-responsive PCa cells undergoes apoptosis in response to ADT, androgen withdrawal invokes a cellular senescence in prostate cancer epithelial cells in vitro and in vivo [72]. ADT-induced senescence led to androgen-refractory behavior in androgen-responsive LNCaP and LAPC4 prostate cancer cells [73]. In these cells, ADT-induced senescence was associated with tumor-promoting features, like chemoresistance and inhibition of p53-mediated cell death, further promoting persistence of the senescent cells [73]. In addition, Perniková et al. showed that ADT-induced senescence was associated with the induction of a tumor-promoting SASP mediated, at least in part, by the down-regulation of Skp2 [74]. More recently, Blute Jr and colleagues showed that senescence is induced preferentially in intermediate-grade versus high-grade cancer, suggesting that senescence could explain the persistence of some PCa cells after ADT in tumors [75]. Altogether, these studies highlight the detrimental roles of long-term presence of senescent cells, suggesting that targeting senescent cells for removal may improve outcomes.

## 4. Senescence in the Bladder

### 4.1. Aging Bladder

The prevalence of bladder dysfunction increases with age and strongly affects the quality of life of both men and women, in addition to prostatic disorders in men. Urodynamic studies have demonstrated advancing age to be associated with reduced bladder capacity, increased uninhibited contractions, decreased urinary flow rate (particularly in men), diminished urethral pressure profile (particularly in women), and increased postvoid residual urine volume [76]. Besides, aging is associated with a thickened bladder muscularis layer, increased frequency of micturition and much higher voiding pressure in rats [77,78]. While voiding dysfunction in the elderly appears to include deterioration of bladder muscle function and increased wall fibrosis, only few studies have focused on the molecular mechanisms underlying aging-related bladder dysfunction. 

Recently, it has been shown that urothelial senescence increased with age in Wistar urothelium [24]. In addition, cultured urothelial cells can undergo a telomere-shortening-independent senescent phenotype, suggesting that senescence could participle in the aging-related bladder alterations [79]. Mechanistically, aging bladder dysfunction was linked to mitochondrial damage-induced NLRP3/IL-1β inflammasome activation [80]. This feature was associated with consequent collagen deposition, p21 increase, and bladder dysfunction in female rats [80].

### 4.2. Pathological Conditions Associated with Bladder Dysfunction

Recently, it has been hypothesized that urothelial senescence could contribute to increased SASP-mediated inflammation and oxidative stress in the bladder wall, in the condition of diabetic bladder dysfunction [24]. Within this line, Klee and colleagues have demonstrated that high glucose levels increased cellular senescence in primary bladder smooth muscle cells [24]. Nevertheless, whether accumulation of senescent cells could participate in bladder dysfunction observed in diabetic patients remains to be determined.

Interestingly, the role of senescence has been more studied in bladder cancer, while providing discrepant results. Indeed, upregulation of a novel senescence gene (SENEX) contributed to regulatory T cells accumulation in aged urinary bladder cancer, further promoting tumorigenesis and metastasis [81]. In addition, expression of senescence markers including p14, p16, p21, and p53 showed a highly-relevant correlation to the pathological outcome of muscle-invasive bladder cancer [82]. It is noteworthy that, while individual expression of these proteins was not related to cancer-specific survival, simultaneous expression of p14 and p53 proteins showed greater clinical relevance. Therefore, patients with p14+/p53- had significantly shorter survival than those with other p14/p53 expression categories [82]. P14 expression, with or without simultaneous p53 expression, was significantly associated with shorter survival in patients treated with adjuvant chemotherapy, indicating that p14 might be involved in tumor response to chemotherapy [82]. Conversely, some bioactive molecules have been used to limit cancer cell proliferation through induction of senescence. Within this line, the O-methylated flavonol isorhamnetin, inhibited human bladder cancer cells proliferation by ROS-dependent arrest of the cell cycle at the G2/M phase [83]. Of note, these effects were associated with an increase in p21 but also induction of apoptosis [83]. In addition, quinone-containing molecules inhibit the proliferation of cancer cells through a mechanism involving a MAPK-dependent senescent phenotype [84]. Overall, these data highlight the tricky role of senescence in bladder cancer progression. Of note, the recent identification of new senescence markers in a bladder cancer cell line may help to elucidate the senescence-associated mechanisms that determine the fate of cancer cells [85].

## 5. Senotherapeutics in Renal and Urinary Tract Disorders

Importantly, genetic mouse models of senescence inhibition, through the use of p16^INK4A^ and p21^CIP1^ knockout mice, have brought substantial insights on the role of senescent cells in renal and urinary tract disorders [35,37,48]. Likewise, other transgenic models have made major contributions to our understanding of the role of senescence, by inducing a selective depletion of cells after they have become senescent. This has been mainly achieved by using the promoter for the p16^INK4A^ gene to drive the expression of a concomitant suicide gene (typically FK506-binding-protein-caspase8), which can subsequently be activated by administration of a drug (AP20817) [86]. These transgenic INK-ATTAC mice exhibited a 75% reduction in the numbers of senescent cells in the kidneys, which was associated with a significant decrease in glomerulosclerosis and blood urea levels [87]. A comparable mouse model (p16::3MR) was developed by Baar and colleagues, where the promoter for the p16^INK4A^ gene drives expression of thymidine kinase from the herpes aimplex virus, which induces apoptosis when cells are presented with the antiviral drug Ganciclovir [88]. Interestingly, p16::3MR mice treated with Ganciclovir displayed lower levels of plasma serum urea and creatinine [88]. 

Since elimination of senescent cells through genetic approaches mitigated aging and renal disorders, pharmacological intervention targeting senescent cells has been proposed. These drugs, named as senotherapeutics, aim at decreasing senescent cell number (senolytics) and/or inhibiting their function (senomorphics/senostatics), and might be highly valuable to facilitate the translation of results from basic research to humans. 

### 5.1. Senolytics

The first class of molecules that effectively target senescent cells are the so-called senolytics. Their goal is either to inhibit senescent cell anti-apoptotic pathways (SCAPs) or to stimulate apoptosis of senescent cells, so as to eliminate them. In this line, a variety of drugs including small molecules, peptides, and antibodies are being developed [89,90,91]. Among them, ABT-263 (navitoclax), FOXO4-DRI, and the combination of dasatinib plus quercetin (D+Q) have been more extensively studied in a variety of models. ABT-263 is an inhibitor of the Bcl-2 family of anti-apoptotic proteins that has shown beneficial effects in murine models of neurodegenerative diseases [92], atherosclerosis [93], or cardiac ischemia–reperfusion [94] through senescent cell clearance. Interestingly, ABT-263 also induced apoptosis of senescent renal epithelial cells, but the impact of ABT-263 on renal function remains to be uncovered [95]. Of note, thrombocytopenia has been reported as a major adverse effect of ABT-263 and constitutes a limitation to the therapeutic potential of the drug, especially when aiming to restore the healthspan of aged individuals [96]. Recently, a cell-penetrating peptide has been designed to disrupt the endogenous p53-FOXO4 interaction, causing nuclear exclusion of active p53 [88]. As a consequence, this peptide (FOXO4-DRI) selectively and potently targets senescent cells for p53-dependent apoptosis. Importantly, FOXO4-DRI counteracted ionizing radiation- and doxorubicin-induced senescence, further preventing loss of liver function [88]. Interestingly, FOXO4-DRI also prevented frailty and loss of renal function in naturally-aged mice and in a murine model of accelerated aging (mimicking the human premature-aging syndrome trichothiodystrophy), by normalizing tubular IL-6 elevation, lowering plasma urea levels, and reducing the levels of senescence markers [88]. Of note, contrary to ABT-263, FOXO4-DRI did not alter platelet levels. Among the other drugs that are being used as senolytics, the combination of dasatinib plus quercetin (D+Q) probably constitutes the most studied ones [97]. Dasatinib is an ATP-competitive protein tyrosine kinase inhibitor used as an antineoplastic drug to treat chronic myeloid leukemia and acute lymphoblastic leukemia. Quercetin is a flavonoid that inhibits PI3K, other kinases, and serpines [97]. D+Q therefore eliminate senescent cells by disabling pro-survival networks, and this combination has proven positive health benefits in various models of aging and age-related dysfunctions [98,99,100]. Regarding renal function, D+Q has led to an improvement of microalbuminuria and renal podocyte function in a model of obesity-induced metabolic dysfunction [17]. In addition, Kim at al. showed that quercetin alone led to improved tubular epithelial cell viability, normalized levels of creatinine and microalbuminuria, and reduced renal cortical hypoxia in HFD mice [101]. Importantly, D+Q is being tested in ongoing clinical trials and the first results showed that the combination is safe and well-tolerated in idiopathic pulmonary fibrosis [102]. Interestingly, the preliminary results of a phase II trial demonstrated that D+Q decreases blood SASP factors, senescent cell abundance, and associated inflammation in adipose tissue of patients with diabetic kidney dysfunction [103]. These encouraging results suggest that the effects observed in mouse models are also observable in humans, opening up therapeutic avenues in the treatment of age-related pathological conditions like renal and urinary tract disorders. Of note, the field of senolytics is rapidly growing and other senolytic molecules have emerged recently [91]. Nevertheless, further investigations are needed to uncover their potential roles in renal and urinary tract disorders.

### 5.2. Senostatics/Senomorphics

In addition to senolytic drugs that aim at clearing senescent cells, other therapeutic options have been shown to modulate the phenotype of senescent cells. They avert senescence by interfering with senescent-associated intracellular pathways, inflammation, and SASP, without induction of senescent cell apoptosis [104]. These senostatic/senomorphic drugs include molecules like p38 inhibitors, Janus kinase (JAK) pathway inhibitors and inhibitors of IkB kinase (IKK) and nuclear factor (NF)-kB [105]. Interestingly, metformin, an oral hypoglycemic agent, has shown promising effects in patients with diabetic nephropathy through inhibition of NF-κB signaling and consequent SASP [106,107]. In addition to the aforementioned drugs, a wide range of previously-described anti-aging compounds have been classified as senostatics/senomorphics candidates. These include telomerase activators, caloric restriction diets, sirtuin activators, antioxidants, anti-inflammatory agents, autophagy and proteasome activators, and mTOR inhibitors [104]. Within this line, the mTOR inhibitor rapamycin reduced the inflammatory and fibrotic responses in the kidneys of mice with unilateral ureteral obstruction [108]. Nevertheless, the potential role of senostatics/senomorphics in lower urinary tract dysfunction is still unknown.

Overall, the field of senotherapies is rising quickly and offers various options to deplete senescent cells or to inhibit their deleterious consequences. Despite some of them are being successfully tested in renal diseases, their benefits in prostate and bladder dysfunctions are still to be assessed. Of note, other strategies relying on immune-system-mediated clearance of senescent cells are emerging and could be an attractive approach for reducing senescent cell burden in renal and urinary tract disorders [109]. 

## 6. Discussion

**Figure 2:**
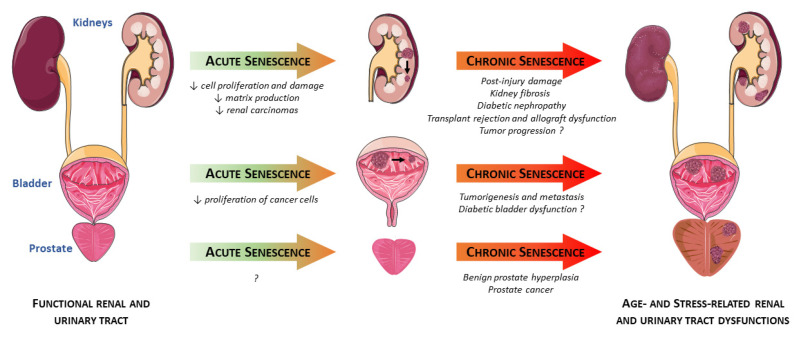
Potential roles of senescent cells in renal and urinary tract disorders. Cellular senescence plays major but intricate roles in the progression of renal and urogenital disorders including kidney, prostate, and bladder dysfunction. While transient senescence appears to be beneficial in acute pathological conditions, chronic senescence hastens urogenital alterations. Senescent cell accumulation is linked to various deleterious conditions like kidney damage and fibrosis, transplant rejection, or benign prostate hyperplasia. Of particular importance, the release of pro-inflammatory and pro-fibrotic mediators by senescent cells is a powerful driver of tumor progression in the bladder and the prostate.

Cellular senescence plays major but intricate roles in the progression of renal and urogenital disorders including kidney, prostate, and bladder dysfunctions and diseases (Figure 2). Indeed, although chronic accumulation of senescent cells hastens post-injury urogenital alterations, transient senescence appears to be beneficial in acute pathological conditions. Activation of senescence seems of great importance in the transitory regulation of cell proliferation and limitation of matrix production, thereby mitigating post-injury renal fibrosis [48]. This dual role of senescence is also well illustrated in urogenital malignancies, where senescence limits proliferation of renal and bladder cancer cells through cell cycle arrest. Nevertheless, long-term presence of senescent cells provides a pro-tumorigenic environment, by releasing pro-inflammatory mediators that are powerful drivers of tumor progression (Figure 2). These observations suggest a complex role of cellular senescence, the latter acting in a stress- and/or time-dependent manner in the progression of lower urinary tract dysfunction and tumorigenesis. In this context, therapeutic interventions with senolytics or senostatics/senomorphics may help elucidating the function of senescent cells and may be beneficial in reversing age- and/or stress-related urogenital disorders. Importantly, several parameters need to be considered to improve the chance of success of senotherapies. Among them, the timing of treatment is determinant as it seems more advantageous in the early stages of dysfunction, when the number of senescent cells is still limited. Therefore, specific removal/inhibition of senescent cells in a well-selected timing may be the most feasible and most attractive approach for clinical application, and senotherapeutic molecules could greatly help in this way to make a fast translation to clinical practice in the field of kidney, prostate, or bladder dysfunctions.
